# Complete Genome Sequence of Porcine Parvovirus N Strain Isolated from Guangxi, China

**DOI:** 10.1128/genomeA.01359-14

**Published:** 2015-01-08

**Authors:** Qian-Lian Su, Bin Li, Wu Zhao, Jia-Xing Liang, Ying He, Yi-Bin Qin, Bing-Xia Lu

**Affiliations:** Guangxi Veterinary Research Institute, Guangxi Key Laboratory of Animal Vaccines and New Technology, Nanning, Guangxi, China

## Abstract

We report here the complete genomic sequence of the porcine parvovirus (PPV) N strain, isolated in 1989 from the viscera of a stillborn fetus farrowed by a gilt in Guangxi, southern China. Phylogenetic analyses suggest that the PPV-N strain is closely related to attenuated PPV NADL-2 strains. The PPV-N strain has good immunogenicity, genetic stability, and safety.

## GENOME ANNOUNCEMENT

Porcine parvovirus (PPV) was discovered by Mary and Mahnel while performing tissue culture of classical swine fever virus in 1966. PPV is one of the main pathogens causing swine reproductive disorders and fetal malformation. PPV can cause infertility, abortion of primiparous sows, mummified fetuses, and stillborn fetuses. The positive detection rate of PPV in pig farms around the world in herds is very high, and it has brought great economic losses to the pig industry (1, 2). PPV, which belongs to the *Parvoviridae* family, is a self-replicating single-stranded DNA virus that has the unique structure of the nucleic acid by folding to form a U-shaped structure at the 5' terminus and a Y-shaped structure at the 3' terminus. The PPV genome encodes three nonstructural proteins, NS1, NS2, and NS3, and two structural proteins, VP1 and VP2; another structural protein, VP3, is derived from the hydrolysis of the VP2 protein (3, 4). Natural attenuated porcine parvovirus N strain (PPV-N strain) was isolated in 1989 from the viscera of a stillborn fetus farrowed by a gilt in southern China (5). Vaccines are important tools in PPV disease prevention. The PPV-N strain was developed for a PPV attenuated vaccine, and animal testing showed that it has good immunogenicity, genetic stability, and safety (6–9).

Referring to the nucleotide sequence of the PPV reference strain published in the GenBank database (GenBank accession no. NC_001718), primers were designed using the Primer Premier 5 software (PREMIER Biosoft International, Inc.). The PCR amplification products were recovered and purified with a gel extraction kit (TIANGEN Biotech Co., Ltd.), and the purified products were T-A cloned into sequencing vector with the pMD18-T vector kit (TaKaRa Biotechnology Co., Ltd.). The recombinant plasmid containing strain PPV-N genomic fragments was sequenced by a commercial sequencing service corporation, a sequencing method was used for the positive reverse bidirectional determination of the nucleotide sequence, and the DNAStar Lasergene 7.1 software (DNAStar, Inc.) was used for splicing and analysis of strain PPV-N genomic fragment nucleotide sequences.

The complete genome of strain PPV-N is 4,853 nucleotides, including the VP1, VP2, NS1, NS2, and NS3 genes, and the lengths of these genes are 2,190, 1,740, 1,989, 486, and 324 nucleotides, respectively. These genes generate capsid protein 1, capsid protein 2, nonstructural protein 1, nonstructural protein 2, and nonstructural protein 3, respectively, and the deduced lengths of these proteins are 730, 580, 663, 162, and 108 amino acids, respectively. The VP1 protein and VP2 protein are PPV capsid proteins, are associated with the antigenicity and virulence of PPV, and play a very important role in the PPV-infected-cell process (10). The VP2 gene sequence determines the phylogeny, hemagglutination activity, tissue tropism, and host range of PPV, and amino acid differences among the dermatitis, attenuated, and virulent strains are located in the coding region of the VP2 gene (11). Using the MEGA 4.1 software, phylogenetic analyses of the VP2 gene of PPV strains revealed that the PPV-N strain is closely related to American attenuated PPV strain NADL-2 (GenBank accession numbers M32787 and M38367) and Canadian attenuated PPV strain NADL-2 (GenBank accession no. NC_001718), suggesting that these viruses are genetically similar (12).

### Nucleotide sequence accession number.

The complete genome sequence of strain PPV-N was deposited in GenBank under the accession no. HM989009.
